# Supplementary material for: Association between type 2 diabetes and osteoporosis risk: A representative cohort study in Taiwan
Source: PLoS One. 2021 Jul 13;16(7):e0254451. doi: 10.1371/journal.pone.0254451 (PMC8277062; doi:10.1371/journal.pone.0254451)
Supplement: S1 Table — (DOCX) [file pone.0254451.s001.docx]

**S1 Table. ICD-9-CM diagnostic codes for osteoporosis**

| **Inclusion criteria** | |
| --- | --- |
| osteoporosis | 733.0, 733.00, 733.01, 733.02, 733.03, 733.09 |
| Vertebral fractures | 805, 805.2, 805.4, 805.6, 805.8, 806, 806.2x, 806.4x, 806.6x, 806.8x |
| Humeral fractures | 812, 812.0x, 812.2x, 812.4x |
| radio-ulnar fractures | 813, 813.0x, 813.2x, 813.4x, 813.8x |
| Femoral neck fractures | 820, 820.0, 820.2, 820.8, 820.0x, 820.2x |
| **Exclusion criteria** | |
| Traffic accidents | E800–E848, E880-E884 |

ICD-9-CM: International Classiﬁcation of Diseases, Ninth Revision, Clinical Modiﬁcation
