# Supplementary material for: Association between type 2 diabetes and osteoporosis risk: A representative cohort study in Taiwan
Source: PLoS One. 2021 Jul 13;16(7):e0254451. doi: 10.1371/journal.pone.0254451 (PMC8277062; doi:10.1371/journal.pone.0254451)
Supplement: S2 Table — (DOCX) [file pone.0254451.s002.docx]

**S2 Table. Anatomical therapeutic chemical codes used to define the medications in the study cohort**

| Medication | Anatomical therapeutic chemical codes |
| --- | --- |
| Anti-osteoporotic agents | SERM, RANKL inhibitor, iPTH, calcitonin, bisphosphonate  IV form: H05AA02,H05BA01,M05BA02,M05BA03,M05BA06,M05BA08,M05BX04  Oral form: G03GB02,G03XC01,G03XC02,L02BA02,M05BA01,M05BA02,M05BA04,M05BA07,M05BB03 |
| Oral corticosteroids | Fludrocortisones, Betamethasone, dexamethasone, methylprednisolone, prednisolone, triamcinolone, hydrocortisone, cortisone, paramethasone, combinations  IV form: H02AB, H02AB01,H02AB02,H02AB04,H02AB06,H02AB08,H02AB09,H02BX  Oral form: H02AA02,H02AB01,H02AB02,H02AB04,H02AB05,H02AB06,H02AB08,H02AB10,H02BX,H02BX91,M01BA03 |
| Anti-hypertensive agents | Beta blocker, α2-agonist, α-blocker, sodium nitroprusside, diuretics , selective aldosterone receptor antagonist, calcium channel blocker, angiotensin-converting enzyme inhibitors, angiotensin receptor blocker, fixed-dose combinations, others (Minoxidil)  C02CA01,C02CA04,C02CA91,C02DB02,C02DC01,C02DD01,C03AA03,C03AA06,C03AA91,C03BA08,C03BA11,C03CA01,C03CA02,C03DA01,C03DA04,C07AA01,C07AA03,C07AA07,C07AA15,C07AB02,C07AB03,C07AB04,C07AB05,C07AB07,C07AB12,C07AG01,C07AG02,C07BB03,C07BB03,C08CA01,C08CA02,C08CA04,C08CA05,C08CA06,C08CA07,C08CA08,C08CA09,C08CA13,C08CA15,C08DA01,C08DB01,C09AA01,C09AA02,C09AA03,C09AA04,C09AA05,C09AA06,C09AA09,C09AA16,C09CA01,C09CA03,C09CA04,C09CA06,C09CA07,C09CA08,C09CA09,C09DA01,C09DA03,C09DA04,C09DA06,C09DA07,C09DA08,C09DA09,C09DX01,C09DX03,C09DX04 |
| Anti-diabetic agents | Insulin, biguanide, sulfonylurea, α-glucosidase inhibitor, thiazolidinedione, glucagon-like peptide 1, sodium glucose co-transporters 2-inhibitor, dipeptidyl peptidase-4 inhibitors, fixed-dose combinations, others (Guar gum, Repaglinide, Nateglinide, Mitiglinide, calcium hydrate)  A10AB01,A10AB04,A10AB05,A10AB06,A10AC01,A10AD05,A10AE04,A10AE05,A10AE06,A10AE54,A10BA02,A10BA03,A10BB01,A10BB02,A10BB03,A10BB05,A10BB07,A10BB08,A10BB09,A10BB12,A10BD,A10BD02,A10BD05,A10BD07,A10BD08,A10BD09,A10BD10,A10BD11,A10BD13,A10BD15,A10BD19,A10BD20,A10BF01,A10BF02,A10BG02,A10BG03,A10BH01,A10BH02,A10BH03,A10BH04,A10BH05,A10BJ01,A10BJ02,A10BJ03,A10BJ05,A10BK01,A10BK02,A10BK03,A10BK04,A10BX01,A10BX02,A10BX03,A10BX08 |
| Lipid-lowering agents | Statins, fibrate, cholestyramine resin, Probucol, Ezetimibe, proprotein convertase subtilisin/kexin type 9 inhibitors, fixed-dose combinations, others (Cholexamin, niacin)  C10AA01,C10AA02,C10AA03,C10AA04,C10AA05,C10AA07,C10AA08,C10AB01,C10AB02,C10AB03,C10AB04,C10AB05,C10AB09,C10AC01,C10AD06,C10AD91,C10AX02,C10AX09,C10AX13,C10BA01,C10BA02,C10BA03,C10BA05 |
