# Supplementary material for: Association between type 2 diabetes and osteoporosis risk: A representative cohort study in Taiwan
Source: PLoS One. 2021 Jul 13;16(7):e0254451. doi: 10.1371/journal.pone.0254451 (PMC8277062; doi:10.1371/journal.pone.0254451)
Supplement: S3 Table — (DOCX) [file pone.0254451.s003.docx]

**S3 Table. The definition of the covariates in the study cohort**

| Covariates | Definition |
| --- | --- |
| Regular exercise habit | Regular exercise more than 30 minutes a day, three times a week, lasting at least three months retrieved from TwSHHH 2002. |
| Current smokers | An adult who has smoked 100 cigarettes in his or her lifetime and who currently smokes cigarettes in recent one month retrieved from 2001 NHIS. |
| Alcohol use almost every day | Alcohol use every day or almost every day retrieved from 2001 NHIS. |
| menopause | Already menopaused retrieved from TwSHHH 2002. |
| Education level | ≥ 9 years of schooling retrieved from 2001 NHIS. |
| Income | Average month income ≥ 40,000 NTD retrieved from 2001 NHIS |
| Living with spouse | Living with spouse retrieved from 2001 NHIS. |
| High calcium diet | Milk or cheese intake every day or almost every day retrieved from 2001 NHIS. |
| Long-term systemic steroid use | Oral corticosteroids use for more than three months between 2002 and 2015 before event day according to the ATC codes. |
| Oophorectomy | Answer “yes” to the question of oophorectomy history in 2001 NHIS |
| Hypertension | Systolic blood pressure ≥ 140mmHg, diastolic blood pressure ≥ 90mmHg in 2002 TwSHHH or prescription of anti-hypertensive agents according to the ATC codes ≥28 tablets one year before the index date. |
| Hyperlipidemia | Low-density lipoprotein cholesterol ≥ 130 mg/dL, triglycerides ≥ 150 mg/dL in 2002 TwSHHH or prescription of the lipid-lowering agents according to the ATC codes ≥28 tablets one year before the index date. |
| Hyperthyroidism | At least twice outpatient diagnosis or once hospitalization discharge diagnosis of ICD9-CM codes for hyperthyroidism (242xx). |

ATC code: anatomical therapeutic chemical code; ICD-9-CM: International Classiﬁcation of Diseases, Ninth Revision, Clinical Modiﬁcation; NHIS: National Health Interview Survey; NTD: New Taiwan Dollars; TwSHHH: Taiwanese Survey on Hypertension, Hyperglycemia, and Hyperlipidemia
