# Supplementary material for: Association between type 2 diabetes and osteoporosis risk: A representative cohort study in Taiwan
Source: PLoS One. 2021 Jul 13;16(7):e0254451. doi: 10.1371/journal.pone.0254451 (PMC8277062; doi:10.1371/journal.pone.0254451)
Supplement: S1 File — (DOCX) [file pone.0254451.s004.docx]

The raw data was owned by the third party: Healthy and Welfare Data Science Center, HWDC

**SAS code**

**data** data.cohort;

set w.h_bhp_twhhh_v2;

array drug(**6**) LIPID_drug DM_drug HTN_drug LIPID_drug_after DM_drug_after HTN_drug_after;

do i=**1** to **6**;

if drug(i)=**.** then drug(i)=**0**;

end;

if age<**40** then delete;   /*age<40 n=3079*/

if pregnant in ("-9999" "-999") then pregnant=**.**;

if gender=**0** and pregnant=**1** then delete;  /*pregnant n=18*/

if dis1_before="1" or dis2_before="1" then delete;  /*n=227*/

if death_day^="." and death_day<=index_day then delete;

/*only for X=DM*/  if type1DM ="1" then delete;  /*n=50 */

/*outcome_py*/

outcome_day=min(d1_day,d2_day,osteo_drug_day,death_day);

if outcome_day=**.**  then outcome_day=mdy(**12**,**31**,**2015**);

outcome_py=(outcome_day-index_day)/**365.25**;

/*outcome*/

if dis1_after=**1** or dis2_after=**1** or osteo_after then outcome=**1**; else outcome=**0**;

if death="." then death=**0**;

if death_day^=**.** and death_day<outcome_day then delete;  /*n=0*/

/**** dis2="nontraumatic fractures" *****/

/*outcome1_py*/

outcome1_day=min(d2_day,death_day);

if outcome1_day=**.**  then outcome1_day=mdy(**12**,**31**,**2015**);

outcome1_py=(outcome1_day-index_day)/**365.25**;

/*outcome1*/

if dis2_after=**1** then outcome1=**1**; else outcome1=**0**;

if death="." then death=**0**;

if death_day^=**.** and death_day<outcome1_day then delete;

/**** outcome but exclude dis2 *****/

/*outcome2_py*/

outcome2_day=min(d1_day,osteo_drug_day,death_day);

if outcome2_day=**.**  then outcome2_day=mdy(**12**,**31**,**2015**);

outcome2_py=(outcome2_day-index_day)/**365.25**;

/*outcome2*/

outcome2=outcome;

if dis2_after=**1** then outcome2=**0**;

if death="." then death=**0**;

if death_day^=**.** and death_day<outcome2_day then delete;

**run**;

**data** data.Cohort_v1;

set data.cohort;

    if n_Glu in ("999" "99.9" "999.9") then n_Glu=" ";

if N_HB in ("999" "99.9" "999.9") then N_HB=" ";

if N_CHOL in ("999" "99.9" "999.9") then N_CHOL=" ";

if  n_tri in ("999" "99.9" "999.9") then n_tri=" ";

if n_hdl in ("999" "99.9" "999.9") then n_hdl=" ";

if n_LDL in ("999" "99.9" "999.9") then n_LDL=" ";

if N_CREAT in ("999" "99.9" "999.9") then N_CREAT=" ";

if n_sgpt in ("999" "99.9" "999.9") then n_sgpt=" ";

if sbp1 in ("999" "99.9" "999.9") then sbp1=**.**;

if sbp2 in ("999" "99.9" "999.9") then sbp2=**.**;

if dbp1 in ("999" "99.9" "999.9") then dbp1=**.**;

if dbp2 in ("999" "99.9" "999.9") then dbp2=**.**;

Glu=n_Glu***1**; HbA1c=N_HB***1**; TC=N_CHOL***1**; TG= n_tri***1**; HDL=n_hdl***1**; LDL=n_LDL***1**; Cr=N_CREAT***1**; GPT=n_sgpt***1**;

/* Waist circumference*/

if **0**<wc_value<**40** then wc_value=wc_value***2.54**;

if WC_value in ("999.9") then WC_value=**.**;

if (gender=**0** AND WC_value>=**80**) or (gender=**1** and WC_value>=**90**) then WC=**1**; else WC=**0**;

/* sbp and dbp */

If sbp1 ne **.** and sbp2 ne **.** then sbp=(sbp1+sbp2)/**2**;

Else if sbp1=**.** Then sbp=sbp2;

Else if sbp2=**.** Then sbp=sbp1;

If dbp1 ne **.** and dbp2 ne **.** then dbp=(dbp1+dbp2)/**2**;

Else if dbp1=**.** Then dbp=dbp2;

Else if dbp2=**.** Then dbp=dbp1;

        if (SBP>=**130** or DBP>=**85**) or HTN_drug=**1** then MSBP=**1**; else MSBP=**0**;

 /* TG */

    if (TG>=**150**) or (LIPID_drug=**1**) then MSTG=**1**; else MSTG =**0**;

 /* HDL */

if (gender=**1** and hdl<**40**) or (gender=**0** and hdl <**50**) then MSHDL=**1**; else MSHDL=**0**;

 /* Glu */

if (Glu>=**100**) or (DM_drug=**1**) then MSglu=**1**; else MSglu=**0**;

/*BMI*/

if BH in ("-999" "999.8") then BH=**.**;

if BW in ("-999" "999.8") then BW=**.**;

if BH=**.** then bh=height2007;

if BH in ("99998" "99999") then BH=**.**;

BMI=BW/((BH/**100**)****2**);

/* BMI 4 groups*/

If **0**<=BMI <**18.5** then BMIc=**1**;

Else If **18.5**<=BMI<**24**  then BMIc=**2**;

Else If **24**<=BMI<**27** then BMIc=**3**;

    Else If **27**<=BMI then BMIc=**4**;

If BMIc=**4** then obe=**1**; else obe=**0**;

/* Metabolic syndrome*/

        if (MSBP+ MSGlu + MSTG +MSHDL + WC)>=**3** then MetS=**1**; else MetS=**0**;

MetS_n=MSBP+ MSGlu + MSTG +MSHDL + WC;

if (MSBP+ MSGlu + MSTG +MSHDL + obe)>=**3** then MetS1=**1**; else MetS1=**0**;

MetS1_n=MSBP+ MSGlu + MSTG +MSHDL + obe;

if (MSBP+ MSGlu + MSTG +MSHDL )>=**2** then MetS2=**1**; else MetS2=**0**;

MetS2_n=MSBP+ MSGlu + MSTG +MSHDL;

/* HbA1C*/

If HbA1c<**7.5** then HbA1cg=**0**;

Else If **7.5**=<HbA1c<**9** then HbA1cg=**1**;

Else If **9**=<HbA1c then HbA1cg=**2**;

/* age*/  If **40**<=age<**65** then agec=**0**; else if age>=**65** then agec=**1**;

/*smoke*/ if (smoke= **4**) and (current_smoke in (**1**,**2**)) then smoker=**1**; else smoker =**0**;

/* Alcohol--alc */  If alcohol="1" then alc=**1**; else alc=**0**;

/*exercise*/  if  PA=**1** then exercise=**1**; else exercise=**0**;

if **1**<=income<=**6** then incomec=**0**;  /*<40000*/

else incomec=**1**; /*>=40000*/

if income in ("-999" "95" "96" "98") then incomec=**0**;

if marriage in (**1**,**6**) then marry=**1**;  /*Living with spouse*/

else marry =**0**; /*single or divorced or seperated*/

if gender=**0** and menopause =**1** then menopausec=**1**; else menopausec=**0**;

if gender=**0** and HRT in ("2" "3") then HRTuse=**1**; else HRTuse=**0**;

If milk in ("1" "2") or cheese in ("1" "2") then highCa=**1**; else highCa=**0**;

if gender=**0** and ovaryresect1 in("2" "3") then ovaryresect=**1**; else ovaryresect=**0**;

if (Glu>=**126** or HbA1c>= **6.5**) or (DM_drug=**1**) then DM=**1**; else DM=**0**;

If (LDL>=**130**) or (TG>=**150**) or (LIPID_drug=**1**) then HL=**1**; else HL=**0**;

if (SBP>=**140**) OR (DBP>=**90**) or HTN_drug=**1** then HTN=**1**; else HTN=**0**;

if Cr <=**0.7** and gender=**0** then eGFR=**144***((Cr/**0.7**)**(-**0.329**))*(**0.993****age);

else if Cr>**0.7** and gender=**0** then eGFR=**144***((Cr/**0.7**)**(-**1.209**))*(**0.993****age);

else if Cr <=**0.9** and gender=**1** then eGFR=**141***((Cr/**0.9**)**(-**0.411**))*(**0.993****age);

else if Cr > **0.9** and gender=**1** then eGFR=**141***((Cr/**0.9**)**(-**1.209**))*(**0.993****age);

if eGFR <**60** then CKD=**1**; else CKD=**0**;

if **10**<=education<=**16** or **91**<=education<=**92** then edu=**1**;

else edu=**0**;

     if education in ("-999" "96") then edu=**.**;

**run**;

/* Table1*/

**proc** **means** n mean std min median max

data=data.Cohort_v1;

var age bmi WC_value sbp dbp Glu TG HDL GPT LDL Cr HbA1c ;

**run**;

**proc** **freq** data=data.Cohort_v1;

table MetS DM

agec gender  BMIc smoker alc exercise  menopausec  marry  incomec  HRTuse highCa  ovaryresect DM HL HTN hyperthyroidism type1DM  hypogonadism POF CLD steroids_after CKD edu obe;

**run**; /*POF=OI=0,   hypogonadism=4*/

**%macro** g1(g1);

ods output ChiSq=chi;

proc freq data=data.Cohort_v1;

table (agec gender  BMIc smoker alc exercise  menopausec  marry  incomec HRTuse highCa  ovaryresect DM HL HTN hyperthyroidism type1DM hypogonadism CLD steroids_after CKD edu obe)*&g1.

/chisq ;

run;

proc means n mean std median min max q1 q3 qrange data=data.Cohort_v1;

class &g1.;

var age bmi WC_value sbp dbp Glu TG HDL GPT LDL Cr HbA1c ;

run;

proc ttest data=data.Cohort_v1;

class &g1.;

var

        age bmi WC_value sbp dbp Glu TG HDL GPT  LDL Cr HbA1c;

run;

**%mend** g1;

%***g1***(MetS);

%***g1***(MetS1);

%***g1***(MetS2);

%***g1***(DM);

/* Table 2 person-year outcome frequency */

**%macro** group(g);

proc means n sum median max Q1 Q3 QRANGE  data=data.Cohort_v1;

class &g. ;

var outcome_py ;

run;

proc freq data= data.Cohort_v1;

table &g.*outcome;

run;

**%mend** group;

%***group***(MetS);

%***group***(MetS1);

%***group***(MetS2);

%***group***(dm);

**proc** **freq** data=data.Cohort_v1;

table MetS_n*MetS;

**run**;

**proc** **freq** data=data.Cohort_v1;

table outcome  outcome1 outcome2

(dis1_after dis2_after osteo_after)*outcome

dis1_after*(dis2_after osteo_after) dis2_after*osteo_after dis2_after*osteo_after*dis1_after;

**run**;

/* Table 2 Cox regression models*/

**%macro** model(m);

data t;

set data.Cohort_v1;

/* Subgroup */

If agec=**1**;

If gender=**1**;

If BMIc=**4**;

If DM=**1**;

    If hba1cg=**2**;

/* Sensitivity */

if outcome_py<=**1** then delete;

proc phreg;

model outcome_py*outcome(**0**) = &m./RL;

run;

 /*X=DM*/

%***model***( DM gender agec)

%***model***( DM gender agec bmic menopausec smoker);

%***model***( DM gender agec bmic menopausec smoker alc exercise);

%***model***( DM gender agec bmic menopausec smoker alc exercise incomec marry edu);

%***model***( DM gender agec bmic menopausec smoker alc exercise incomec marry edu Cr GPT  HTN HL);

%***model***( DM gender agec bmic menopausec smoker alc exercise incomec marry edu Cr GPT  HTN HL highCa HRTuse);

%***model***( DM gender agec bmic menopausec smoker incomec edu marry highCa hyperthyroidism steroids_after ovaryresect CLD  HRTuse);

/* model 2*/

%***model***(DM gender agec bMic smoker alc exercise menopausec marry edu incomec)

/*Full model*/

%***model***(DM gender agec bmic menopausec smoker incomec edu marry alc exercise highCa hyperthyroidism steroids_after ovaryresect CLD HRTuse Cr HTN HL);

 /*p for interaction*/

%***model***(DM gender agec bmic menopausec smoker incomec edu marry alc exercise highCa hyperthyroidism steroids_after ovaryresect CLD HRTuse Cr HTN HL);

%***model***(DM gender agec bmic menopausec smoker incomec edu marry alc exercise highCa hyperthyroidism steroids_after ovaryresect CLD HRTuse Cr HTN HL DM*agec);

%***model***(DM gender agec bmic menopausec smoker incomec edu marry alc exercise highCa hyperthyroidism steroids_after ovaryresect CLD HRTuse Cr HTN HL DM*gender);

%***model***(DM gender agec bmic menopausec smoker incomec edu marry alc exercise highCa hyperthyroidism steroids_after ovaryresect CLD HRTuse Cr HTN HL DM*bmic);

%***model***(DM gender agec bmic menopausec smoker incomec edu marry alc exercise highCa hyperthyroidism steroids_after ovaryresect CLD HRTuse Cr HTN HL DM*Hba1c);

/* check PH assumption by time-dependent variables*/

**proc** **phreg** data=data.Cohort_v1;

  model outcome_py*outcome(**0**) = dm  dm_1t;

  dm_1t = dm*log(outcome_py);

  proportionality_test: test dm_1t;

**run**;

/* log rank test and log log plot */

**proc** **lifetest** data=data.Cohort_v1  plot=(s, lls) ;

  time outcome_py* outcome (**0**);

  strata DM;

**run**;

**data** t;

set data.Cohort_v1;

keep DM outcome outcome_py;

**run**;

**PROC** **EXPORT** DATA= WORK.t

            OUTFILE= "C:\Users\H108044\Desktop\dm.csv"

            DBMS=CSV REPLACE;

**run**;
